# A case of repeat hepatectomy for liver metastasis from solid pseudopapillary neoplasm of the pancreas: a case report

**DOI:** 10.1186/s40792-021-01134-6

**Published:** 2021-03-01

**Authors:** Atsushi Morito, Kojiro Eto, Kozue Matsuishi, Hirokazu Hamasaki, Keisuke Morita, Satoshi Ikeshima, Kei Horino, Shinya Shimada, Hideo Baba

**Affiliations:** 1 Department of Surgery, Japan Community Health Care Organization Kumamoto General Hospital, 10-10 Tori-machi, Yatsushiro, Kumamoto 866-8660 Japan; 2 Department of Gastroenterological Surgery, Graduate School of Medical Sciences, Kumamoto University, 1-1-1 Honjo, Chuo-ku, Kumamoto, 860-8556 Japan

**Keywords:** Solid pseudopapillary neoplasm, Pancreas, Liver metastasis, Repeat hepatectomy

## Abstract

**Background:**

Solid pseudopapillary neoplasm of the pancreas is a rare tumor in young women, metastasizing in only 5–15% of cases, and most commonly to the liver. Although treatment guidelines have not been established, surgical resection is usually performed. We report a rare case of repeat hepatectomy for liver metastases after distal pancreatectomy with solid pseudopapillary neoplasm.

**Case presentation:**

The patient was a 71-year-old woman who underwent distal pancreatectomy for solid pseudopapillary neoplasm, and liver metastasis occurred 4 years after the first surgery. Partial liver resection was performed for four liver metastases, and histopathological examination revealed a diagnosis of liver metastasis from solid pseudopapillary neoplasm. However, 18 months later, liver metastases were detected again; three tumors were identified, and partial resection was performed, which has provided 18 months’ recurrence-free survival.

**Conclusions:**

Long-term prognosis can be expected following R0 resection for resectable liver metastasis from solid pseudopapillary neoplasm.

## Background

Solid pseudopapillary neoplasm (SPN) of the pancreas is a rare tumor that typically occurs in young women, accounting for only 1–2% of pancreatic tumors [1]. Since Frantz first reported SPN in 1959, the number of reported cases has increased [2]. The clinicopathologic features of SPN are unique: slow-growing, low-grade malignancy [3]. SPN metastasizes in only 5–15% of all cases, and common sites include the liver, spleen, omentum, peritoneum, duodenum [4], 5. Surgical resection is considered the most efficient treatment option for SPN. However, the management of metastatic tumors of SPN is unclear. We report a case of repeat hepatectomy for liver metastasis from SPN.

## Case presentation

**Fig. 1:**
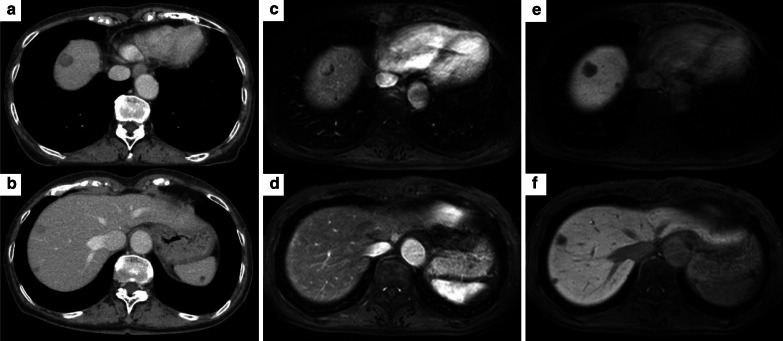
**a**, **b** CT image. Four tumors are seen in liver segments S7 and S8. **c**–**f** EOB-MRI image. The tumor edges are hyperintense with dynamic imaging, and some enhancement is exhibited within the tumors

**Fig. 2:**
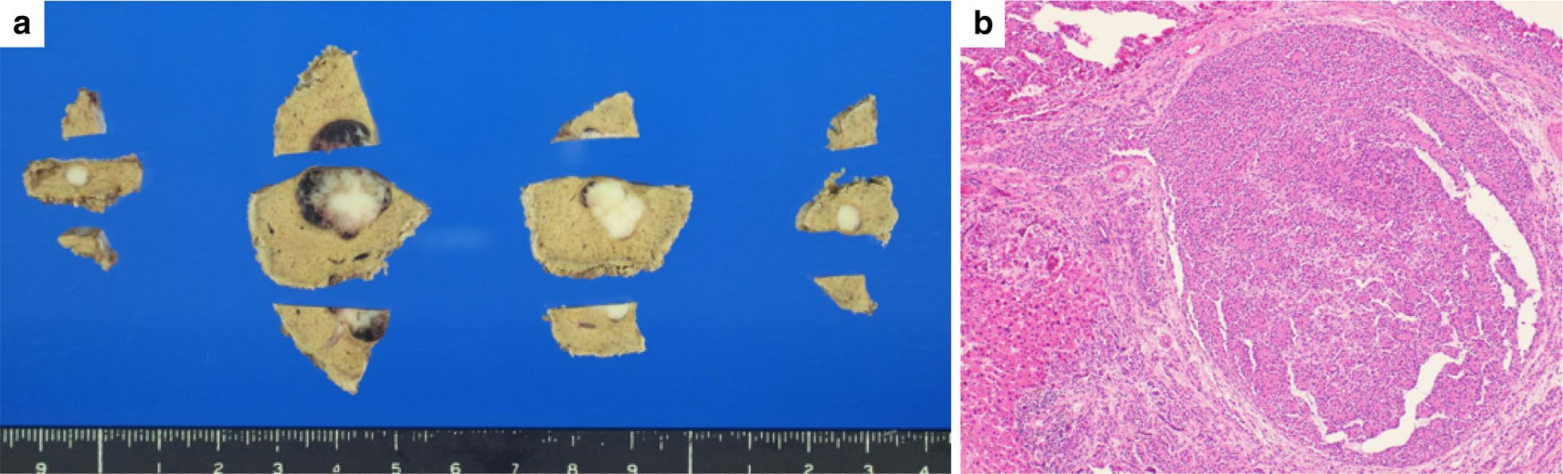
Gross and histopathological findings of the excised specimens. Left image: gross tumor specimens. Right image: photomicrograph showing eosinophilic tumor cells proliferating throughout. The image shows a pseudopapillary structure centered on a thin vascular connection network. These findings are similar to the previous findings

**Fig. 3:**
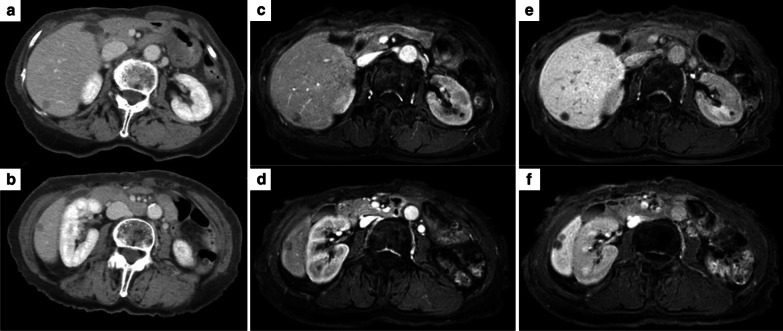
**a**, **b** CT image. Three tumors are visible in liver segments S7 and S8. **c**–**f**: EOB-MRI image. The tumor edges are hyperintense with dynamic imaging, and some enhancement is exhibited within the tumors, similar to the previous findings

**Fig. 4:**
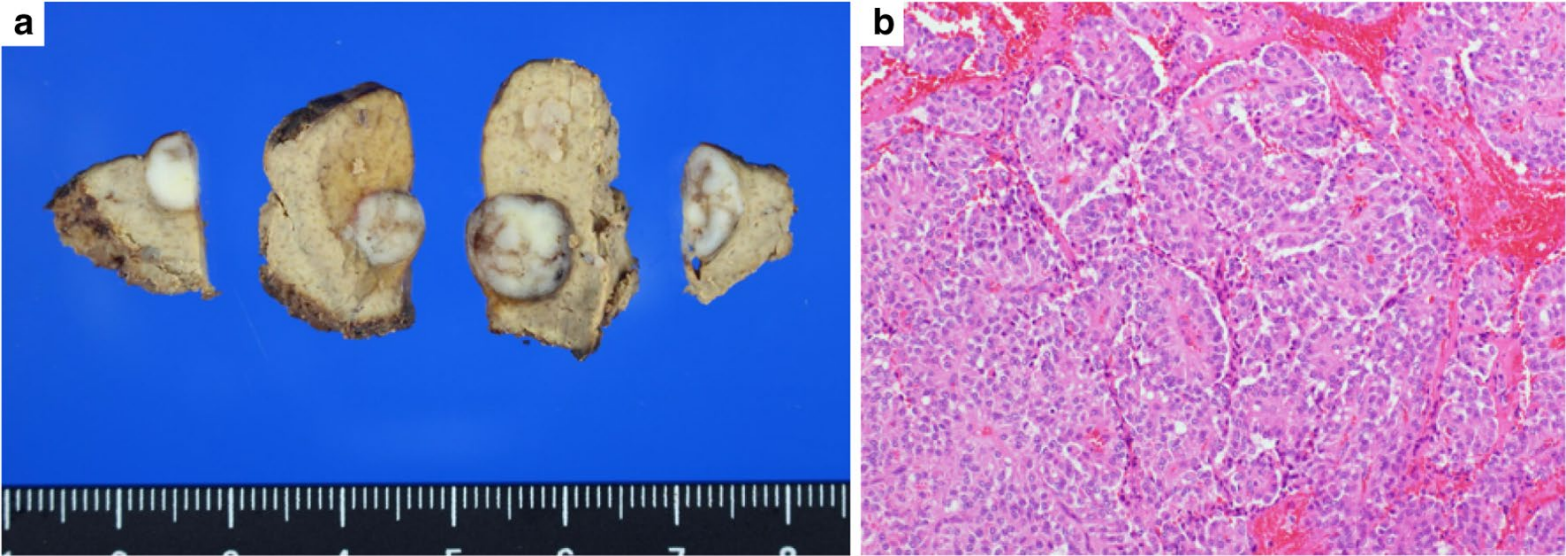
Gross and histopathological findings of the excised specimens. Left image: gross tumor specimens. Right image: photomicrograph showing eosinophilic tumor cells proliferating throughout. The image shows a pseudopapillary structure centered on a thin vascular connection network. These findings are similar to the previous findings

A 71-year-old woman underwent distal pancreatectomy without lymph node dissection for SPN of the pancreas in 2013. The histopathological diagnosis showed solid diffuse growth of circular eosinophilic tumor cells with cystic and hemophilic changes on pseudopapillary structure. In addition, it was partially necrotic and bleeding. Immunohistostaining studies revealed that the tumor cells were positive for vimentin, synaptophysin, cluster of differentiation (CD) 56, β-catenin, CD10, and progesterone receptor. In 2017 (4 years from the first surgery), computed tomography (CT) identified four nodules in the anterior and posterior segments of the right lobe of the liver measuring approximately 15 mm × 14 mm, 12 mm × 11 mm, 8 mm × 8 mm, and 5 mm × 4 mm (Fig. 1a, b); all had low-density areas. Abdominal magnetic resonance imaging (MRI) confirmed the four nodules and revealed hypointensity in the hepatobiliary phase (Fig. 1c–f). Routine laboratory data, including tumor markers, were within normal ranges. Because these tumors were suspected metastases from SPN according to the imaging findings, the patient underwent partial hepatectomy. Pathological examination of the tumors revealed solid growth of circular eosinophilic tumor cells with cystic and hemophilic changes (Fig. 2a, b). In addition, the tumor embolization into the portal vein was observed. Immunohistochemical studies revealed that the tumor cells were positive for vimentin, synaptophysin, CD56, β-catenin, CD10, and progesterone receptor. This was the same result as the first surgery. The Ki-67 labeling index was 3%. These findings closely resembled the initial surgical pancreatic SPN, and we diagnosed SPN metastases. In 2018 (18 months from the first liver resection), we detected recurrent liver metastases. CT identified three low-density areas in the right liver lobe measuring approximately 10 mm × 9 mm, 9 mm × 8 mm, and 6 mm × 6 mm (Fig. 3-a and b). MRI confirmed the tumors (Fig. 3c–f). Because the imaging findings were the same as the previous findings, we considered the new tumors to be liver metastasis from SPN, and the patient underwent partial re-hepatectomy. The pathological and immunohistochemical examination results were the same as those obtained previously (Fig. 4a, b). The patient has remained disease-free 18 months after the re-hepatectomy.

## Conclusions

SPN of the pancreas is a rare neoplasm with low-grade malignant potential [3]. Immunopathological examination is useful for diagnosing SPN. Most pancreatic SPNs are strongly positive for CD10 (96%), progesterone receptor (79%), cytokeratin (28%), synaptophysin (26%), and chromogranin (15%) [6]. This case was positive for CD10 and progesterone receptor; therefore, the diagnosis was liver metastasis from SPN.

Although SPN has low malignant potential, 5%–15% of SPN patients develop metastasis [4] [5] [7]; most commonly to the liver [8]. The number of cases is small, and treatment guidelines have not been established. There are reports of the treatment of liver metastases using chemotherapy [9] [10], transarterial chemoembolization (TACE) [11] [12], and radiofrequency ablation (RFA) [13] [14]. However, these treatments are options only for unresectable cases. In resectable cases, hepatectomy is considered more likely to lead a cure. In our case, the extent of resection was not wide, and liver function was maintained; hepatectomy resulted in an 18-month recurrence-free survival.

Diffuse growth, venous invasion, nuclear pleomorphism, mitotic rate, necrosis and dedifferentiation are histopathological findings suggesting high malignancy of SPN [15]. This case also showed diffuse growth and necrosis at the time of the first surgery, and that may have had a highly malignant tumor. For highly malignant SPN, resection of the metastasis site may lead to a cure.

It is necessary to select treatment considering the tumor malignancy, resection range, liver function, and the timing of the surgery.

In conclusion, we experienced a case of repeat hepatectomy for liver metastasis from SPN. 18-month recurrence-free survival was achieved by surgery.

## Data Availability

All data generated or analyzed during this study are included in this published article.

## Abbreviations

SPN: Solid pseudopapillary neoplasm
CT: Computed tomography
MRI: Magnetic resonance imaging
CD: Cluster of differentiation
TACE: Transarterial chemoembolization
RFA: Radiofrequency ablation
